# Expression of the RNA methyltransferase Nsun5 is essential for developing cerebral cortex

**DOI:** 10.1186/s13041-019-0496-6

**Published:** 2019-08-28

**Authors:** Peipei Chen, Tingting Zhang, Zihao Yuan, Bin Shen, Ling Chen

**Affiliations:** 1 State Key Laboratory of Reproductive Medicine, Nanjing Medical University, Tianyuan East Road 818, Nanjing, China; 2 Department of Physiology, Nanjing Medical University, Tianyuan East Road 818, Nanjing, China

**Keywords:** Cerebral cortex, Migrating neurons, Nsun5, Radial glial cells (RGCs), Williams-Beuren syndrome (WBS)

## Abstract

*Nsun5* gene, encoding a cytosine-5 RNA methyltransferase, is deleted in about 95% patients with Williams-Beuren syndrome (WBS). WBS is a neurodevelopmental disorder and characterized by cognitive disorder. We generated single-gene *Nsun5* knockout (*Nsun5*-KO) mice and reported that the Nsun5 deletion leads to deficit in spatial cognition. This study focused on investigating the influence of Nsun5 deficiency in the development of cerebral cortex. In comparison with wild-type littermates, the cortical thickness in postnatal day 10 *Nsun5*-KO mice was obviously reduced with an abnormal laminar organization, and the processes of pyramidal cells were shorter and finer. Nsun5 was selectively expressed in radial glial cells (RGCs) of cerebral cortex from embryonic day (E) 12.5 to E16.5, but not in intermediate progenitor cells (IPCs) or neocortical neurons. The Nsun5 deletion did not alter proliferation of RGCs or differentiation of RGCs into IPCs. Notably, the ablation of Nsun5 disrupted the growth of radial glial scaffolds, thus numerous basal processes of RGCs failed to reach pial basement membrane. Level of cell polarity regulator Cdc42 protein in radial glial scaffolds of E14.5 *Nsun5*-KO mice was reduced, but the level of *Cdc42* mRNA was unchanged. The dysfunction of glial scaffolds impeded the radial migration of upper-layer and deeper-layer neurons to cause their subcortical accumulation and apoptosis, resulting in an obvious thinness of the cortical plate in E18.5 *Nsun5*-KO mice. These findings establish a critical role of Nsun5 in development of cerebral cortex through regulating radial glial scaffolds of RGCs to control migration of neocortical neurons.

**Electronic supplementary material:**

The online version of this article (10.1186/s13041-019-0496-6) contains supplementary material, which is available to authorized users.

## Introduction

Williams-Beuren syndrome (WBS) is a contiguous gene deletion disorder [1] that is caused by spontaneous deletions of 1.5 million to 1.8 million base pairs comprising 26 to 28 genes on human chromosome 7q11.23 [2]. Using probes containing parts of the elastin gene, the deletion has readily been detected in approximately 90–99% WBS patients [3].

WBS is characterized by an unusual cognitive profile that includes relatively preserved expressive language, facial processing abilities and dramatic deficits in spatial cognition [4–6]. Processing of spatial navigational information and long-term memory, domains highly dependent on hippocampal and cortical function, are also severely affected in WBS [7]. In mice, the entire region of the WBS deletion is conserved on chromosome band 5G2 in a reverse orientation to the centromere and the flanking genes [8]. Most of the genes affected by the WBS deletion are expressed in the brain. Several mouse models have been generated by the single-gene knockout of deleted WBS loci, but relevant phenotypes of neuro-cognitive features are only evident in three heterozygous *Cyln2, Gtf2i, and Gtf2ird1* knockout mouse models [9–11].

The *Nsun5* gene, which encodes a cytosine-5 RNA methyltransferase, is included in the WBS deletion [2, 12]. The deletion of Nsun5 has been reported in about 95% patients with WBS [2]. Zhang et al. have recently reported that single-gene *Nsun5* knockout homozygous (*Nsun5*-KO) mice show the phenotype of spatial cognitive defects [13]. The whole brain volume in patients with WBS has been reported to be reduced by 13% [14]. Mice with partial (approximately half) deletions of the WBS loci showed reductions in brain weight [15] or brain size [16]. Human Nsun5 is reported to co-precipitate with ribosomes [17]. In yeast cells, Nsun5 has been found to directly methylate cytosine 2278 (C2278) of 25S rRNA [18, 19]. The lack of this methylation step leads to a decrease in translational fidelity and a increase in the recruitment of stress-specific mRNAs to translating ribosomes [20]. A loss-of-function mutation in human NSUN2, which encodes a tRNA methyltransferase, causes neurodevelopmental defects including microcephaly [21, 22]. *Nsun2* knockout in mice causes a neuro-cognitive defect [23]. The *Nsun5* transcript is enriched in the developing mouse brain [24]. However, it remains unknown whether Nsun5 deletion affects the development of brain.

The mammalian cerebral cortex is a well-organized structure containing layer-specific classes of neurons and glial cells [25]. The cortical neurons and glia arise from a small heterogeneous population of neural progenitor cells, including radial glial cells (RGCs) in ventricular zone (VZ) and intermediate progenitor cells (IPCs) in subventricular zone (SVZ). RGCs produce both upper-layer neurons and deeper-layer neurons directly or indirectly through IPCs [26]. Thus, the transition from RGCs to IPCs plays a key role in determining the total number of neurons [27]. In developing cerebral cortex, the glial scaffold and molecular polarity of RGCs serve as a critical migratory guide for upper-layer neurons and deeper-layer neurons.

The present study focused on investigating the effects of Nsun5 deficiency on the development of cerebral cortex and the underlying mechanisms. Our results showed that Nsun5 was selectively expressed in RGCs during the developing cerebral cortex. The Nsun5 deficiency impaired the glial scaffold and polarity of RGCs to impede migration of neocortical neurons, which disturbed the laminar organization of neocortical neurons and the development of pyramidal cells.

## Results

### Generation of *Nsun5*-KO mice

**Fig. 1:**
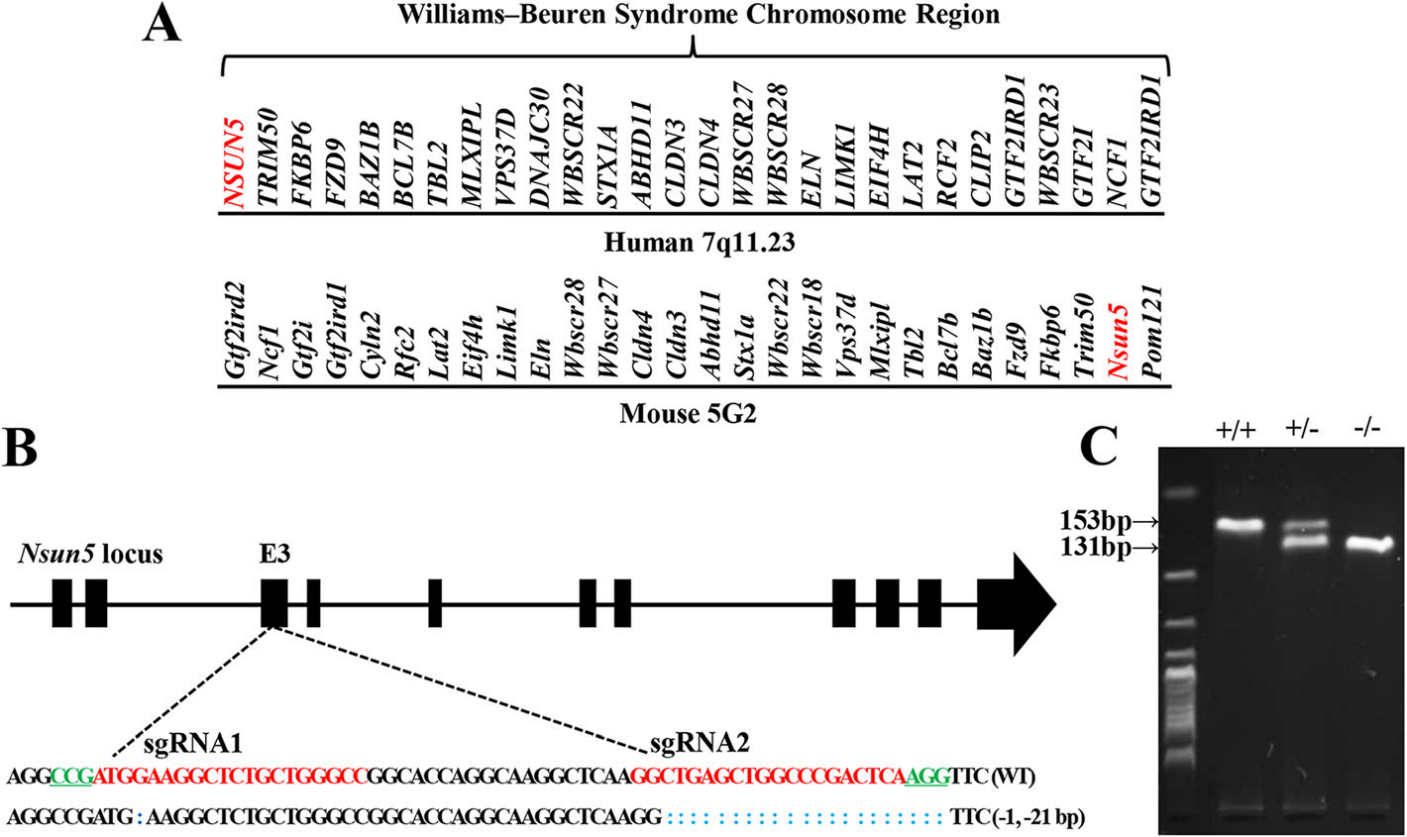
Construction and genotype verification of Nsun5 null mice. **a** Comparison of WBS genomic regions in human and mouse. NSUN5 located in human chromosome 7q11.23 (upper) and mouse chromosome 5G2 (bottom). **b** Two sgRNAs designed to target exon 3 of the *Nsun5* gene. **c** Identification of *Nsun5* DNA in WT mice (+/+), heterozygous (+/−) and homozygous (−/−) mice

In comparison with WBS genomic region in human, the entire WBS deletion gene order in mouse shows a reverse orientation (Fig. 1a). The *Nsun5* gene has been determined to include in the 28 genes of WBS deletion [2, 12]. To investigate the influence of Nsun5 deficiency in the development of cerebral cortex, we used CRISPR/Cas9 genome editing technique to generate the *Nsun5*-KO mouse as previously described [28]. Two sgRNAs were designed to target exon 3 of the *Nsun5* gene (Fig. 1b). The oligos used to generate sgRNA expression plasmids were annealed and cloned into the BsaI sites of pUC57-sgRNA (Addgene 51,132). The genotype was determined by PCR using the genomic DNA obtained from tail biopsies. The *Nsun5* deletion in homozygous (−/−) mice was determined by the amplification of 131 bp fragment and the loss of 153 bp fragment (Fig. 1c).

### Nsun5 deficiency impairs development of cerebral cortex

**Fig. 2:**
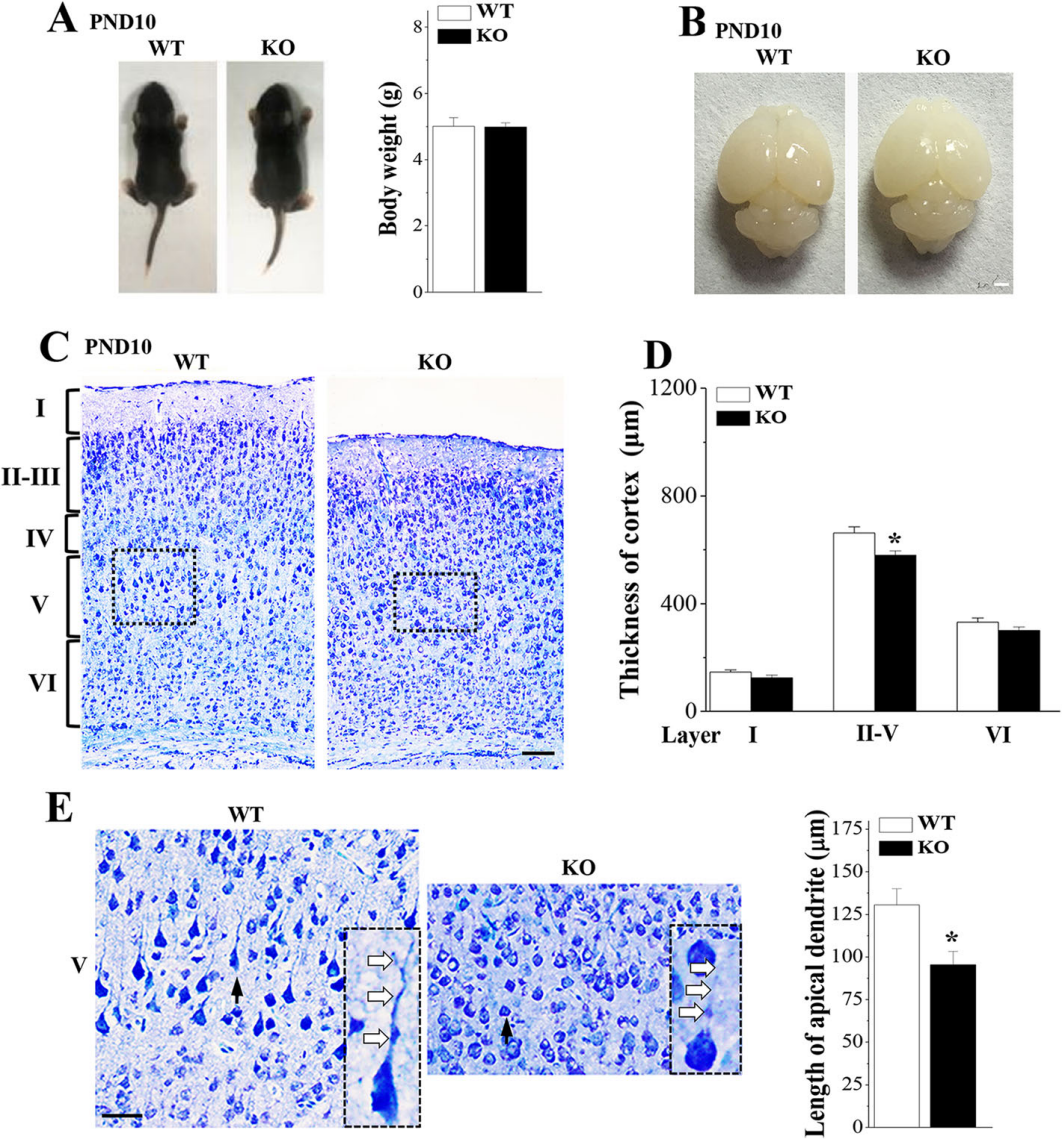
Nsun5 deficiency impairs development of cerebral cortex. **a** & **b** Body weights and pictures of entire brain of PND10 WT mice (WT) and *Nsun5*-KO mice (KO). Scale bars, 1 mm. **c** Representative images of cerebral cortex stained with toluidine blue. Scale bars, 100 μm. **d** Bar graph shows the thickness of layers I-VI. *P < 0.05 vs. WT mice (Student’s t test). **e** Higher power views of the boxed areas in Fig. **c**. Scale bars, 50 μm. Representative images of pyramidal cells (arrows) are shown in the bottom right insets. The open arrows indicate the processes and branches of pyramidal cells. Bar graph shows the length of the apical dendrite of pyramidal cells in the layer V. *P < 0.05 vs. WT mice (Student’s t test)

*Nsun5*-KO newborn pups were survived and their body weights did not differ significantly from those of wild-type (WT) littermates (*P* > 0.05, *n* = 12 mice per experimental group; Fig. 2a). As shown in Fig. 2b, the size of entire brain in postnatal day (PND) 10 *Nsun5*-KO mice was not obviously different from the age-matched WT mice (*n* = 6). However, the thickness of the cortical plate in *Nsun5*-KO mice was obviously reduced compared with WT mice (*P* < 0.05, *n* = 6, Fig. 2c). It is mainly because of the thinning of layers II-V (*P* < 0.05, *n* = 6, Fig. 2d). Notably, the length of the apical dendrite of pyramidal cells in the layer V of *Nsun5*-KO mice were finer (Fig. 2e) and shorter than those in WT mice (*P* < 0.05, *n* = 6).

### Selective expression of Nsun5 in RGCs of developing cerebral cortex

**Fig. 3:**
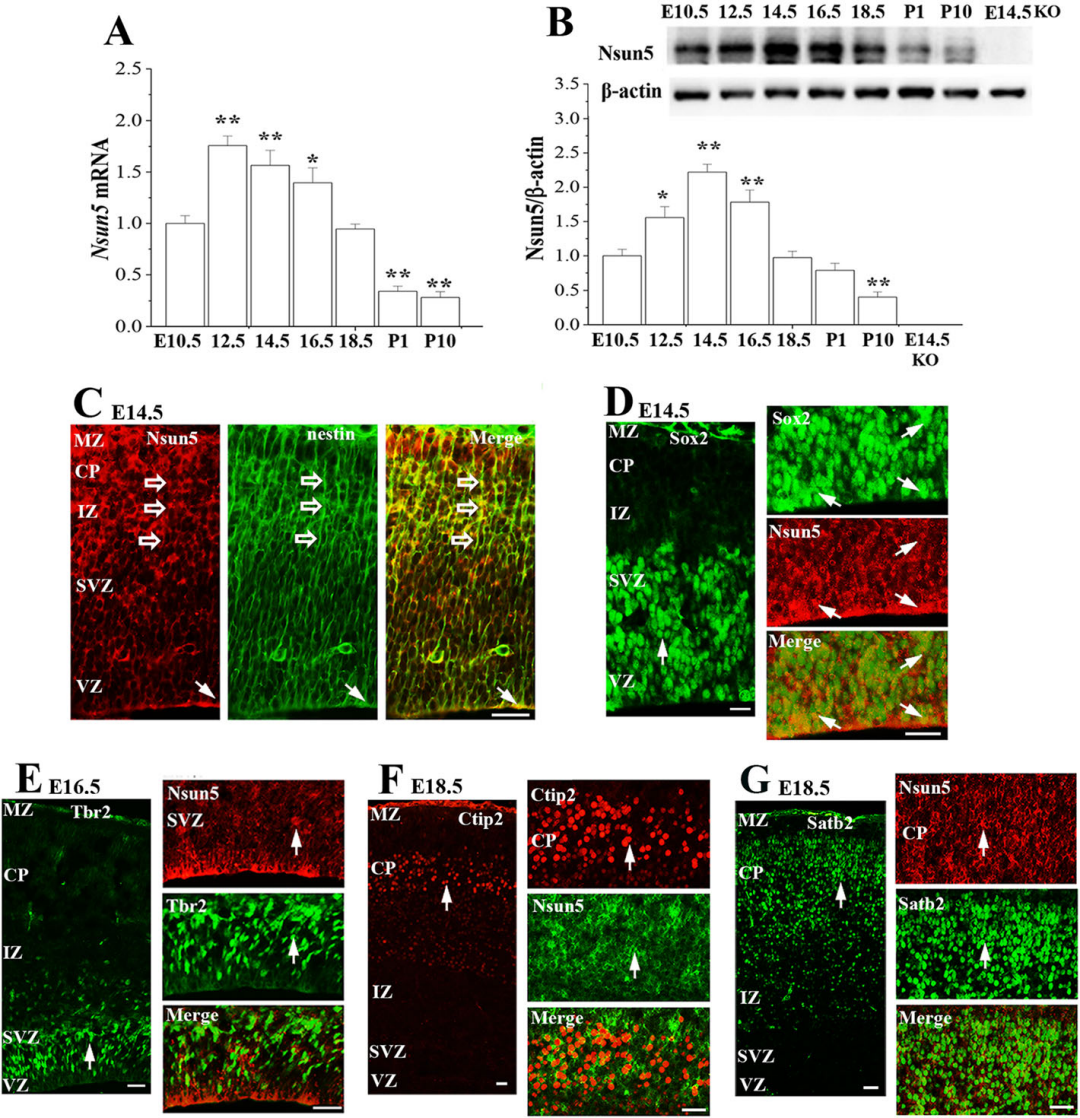
Dynamic expression of Nsun5 during corticogenesis. **a** & **b** Bar graphs show the levels of cerebral cortex *Nsun5* mRNA and Nsun5 protein in E10.5, E12.5, E14.5, E16.5, E18.5, PND1 (P1) and PND10 (P10) WT mice, and E14.5 *Nsun5*-KO mice (KO). *P < 0.05 and **P < 0.01 vs. E10.5 mice (one-way ANOVA). **c** Representative images of double immunostaining in cerebral cortices from WT mice for (**c**) Nsun5 (red) and nestin (green) at E14.5, Scale bars, 25 μm; **d** Sox2 (green) and Nsun5 (red) at E14.5, Scale bars, 25 μm (views of entire cortex); Scale bars, 25 μm (in VZ); **e** Nsun5 (red) and Tbr2 (green) at E16.5, Scale bars, 25 μm (views of entire cortex); Scale bars, 25 μm (in SVZ); **f** Nsun5 (green) and Ctip2 (red) at E18.5, Scale bars, 25 μm (views of entire cortex); Scale bars, 25 μm (in CP); **g** Nsun5 (red) and Satb2 (green) at E18.5, Scale bars, 25 μm (views of entire cortex); Scale bars, 25 μm (in CP). Open arrows represent Nsun5+/nestin+ basal processes of RGC (**c**); arrows indicate Sox2+ cells (**d**), Tbr2+ cells (**e**), Ctip2+ cells (**f**) and Satb2+ cells (**g**)

To explore the mechanisms underlying Nsun5 deletion-impaired development of cerebral cortex, we primarily examined the expression of Nsun5 in cerebral cortex of WT mice from E10.5 to PND10 by RT-PCR and Western blotting (*n* = 6 mice per experimental group). Notably, the levels of the *Nsun5* mRNA (vs. E10.5, *P* < 0.01, *n* = 6, Fig. 3a) and Nsun5 protein (vs. E10.5, *P* < 0.05, *n* = 6, Fig. 3b) were obviously elevated starting from E12.5. However, the time of Nsun5 protein reaching peak stage had a clear lag in comparison with the level of *Nsun5* mRNA. The highest levels of the *Nsun5* mRNA were determined at E12.5 (*P* < 0.01, *n* = 6), while the level of Nsun5 protein reached peak at E14.5 (*P* < 0.01, *n* = 6), followed by a decrease from E18.5, showing transiently high expression of Nsun5 in the developing cerebral cortex.

Subsequently, the double immunostaining for Nsun5 with antibodies against Sox2 or nestin (markers of RGCs), Tbr2 (a marker of IPCs), or Ctip2 and Satb2 (markers of deeper-layer and upper-layer neurons, respectively) were performed to identify the cells expressing Nsun5. At E14.5, the nestin positive (nestin+) processes extended radially throughout the cerebral wall (Fig. 3c) and overlapped with Nsun5 immunoreactivity. As shown in Fig. 3d, Sox2 positive (Sox2+) cells in the VZ of cerebral cortex (left panel) expressed the Nsun5 protein (right panel). In contrast, the Nsun5 protein was not detected in Tbr2 positive (Tbr2+) cells in the SVZ (Fig. 3e). In E18.5 CP, neither the Ctip2 positive (Ctip2+) cells (Fig. 3f) nor the Satb2 positive (Satb2+) cells (Fig. 3g) showed Nsun5 immunoreactivity.

### Nsun5 deficiency disrupts growth of radial glial scaffold

**Fig. 4:**
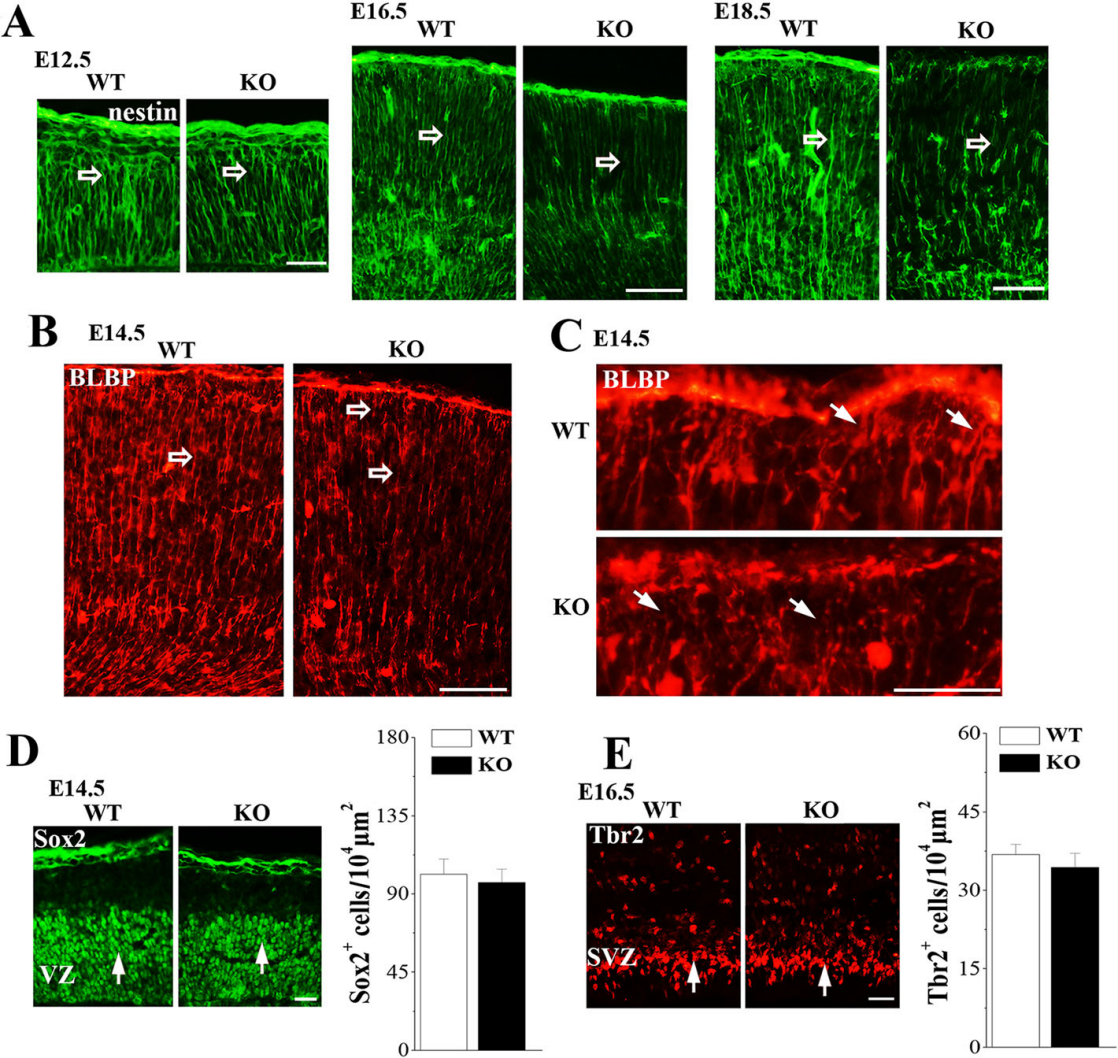
Nsun5 deficiency disrupts the radial glial scaffolds. **a** & **b** The development of radial glial scaffolds was compared between E12.5-E18.5 WT mice and *Nsun5*-KO mice. Images of nestin (green) or BLBP (red) immunostaining. Scale bars, 50 μm (**a**); 25 μm (**b**). Many long, parallelly distributed nestin+ or BLBP+ processes span throughout the cortical wall in WT mice. In E14.5-E18.5 *Nsun5*-KO mice, the radial processes are discontinuous and shorter (open arrows). **c** High magnification images of BLBP labeling at E14.5. Scale bars, 10 μm. In WT mice the end-feet of RGC scaffolds attached to the pial BM, but in *Nsun5*-KO mice many RGC scaffold end-feet detached from the pial BM (arrows). **d** & **e** Representative images of Sox2 at E14.5 or Tbr2 at E16.5. Scale bars, 25 μm. Bar graphs show the numbers of Sox2+ cells and Tbr2+ cells in WT mice and *Nsun5*-KO mice

Above observations determined that RGCs selectively expressed Nsun5 during developing cerebral cortex. Thus, we examined influence of the Nsun5 deletion in the development of RGCs and the radial glial scaffolds of RGCs by the immunohistochemistry for nestin, brain lipid binding protein (BLBP) and Sox2. A short apical process anchors the RGC soma at the ventricular surface, while their long basal processes extend to the pial surface [29, 30]. As shown in Fig. 4a, the nestin+ basal processes radially spanned the entire cerebral cortex in E16.5 and E18.5 WT mice. In contrast, the density of basal processes in *Nsun5*-KO mice was distinctly reduced, and numerous short basal processes failed to extend perpendicularly and reach the pial basement membrane. The histological differences were readily discernible in *Nsun5*-KO mice beginning at E14.5, an earlier stage of RGCs scaffold, because the density of BLBP positive (BLBP+) glial scaffolds in E14.5 *Nsun5*-KO mice was substantially reduced (Fig. 4b), and the basal processes emanating from the VZ were discontinuous. Moreover, few basal end-feet reached the pial surface to interact with the pial basement membrane in E14.5 *Nsun5*-KO mice (Fig. 4c). However, the number of Sox2+ cells in E14.5 *Nsun5*-KO mice was approximately equal to WT mice (*P* > 0.05, *n* = 6, Fig. 4d). In addition, the number of Tbr2+ IPCs within the SVZ was not significantly different between E16.5 WT mice and *Nsun5*-KO mice (*P* > 0.05, *n* = 6, Fig. 4e).

### Nsun5 deficiency impedes the radial migration of cortical neurons

**Fig. 5:**
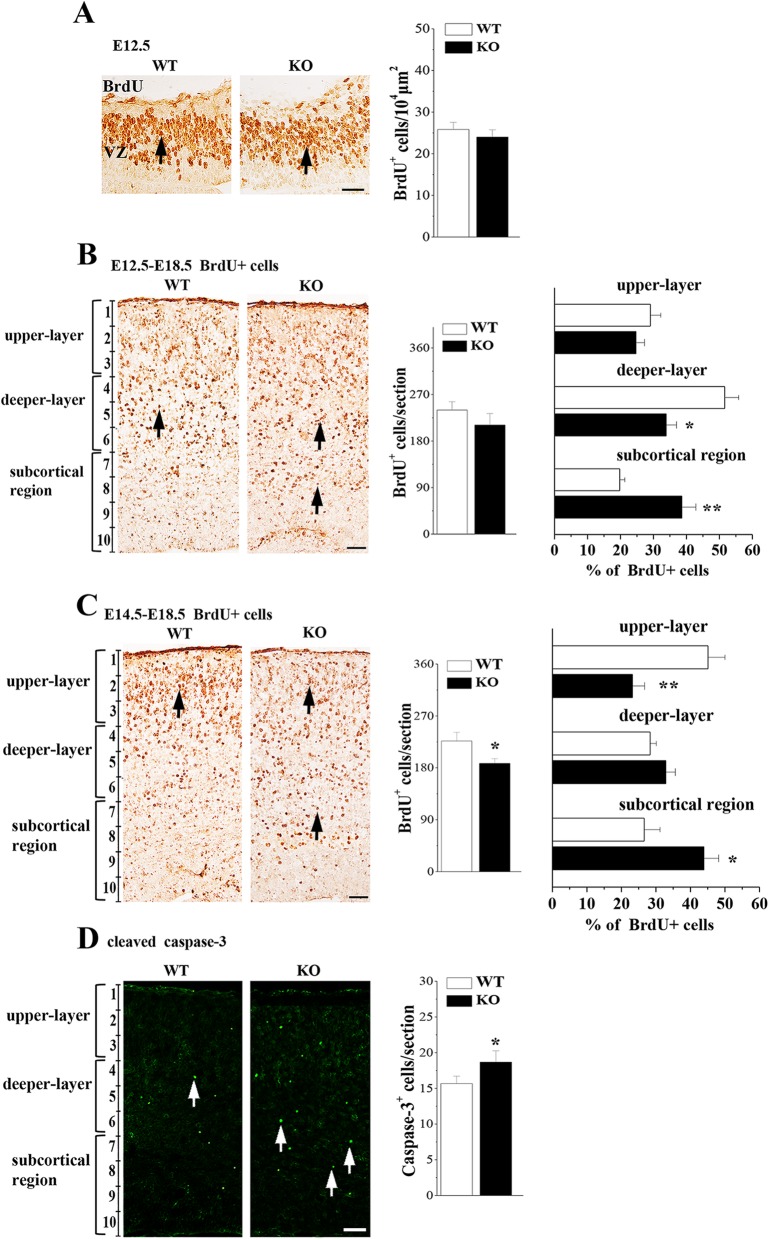
Nsun5 deficiency affects the radial migration of cortical neurons. **a** Representative images of BrdU immunostaining at E12.5, arrows indicate BrdU+ cells, Scale bars, 25 μm. Bar graphs show the numbers of BrdU+ cells. **b** & **c** The cortical distribution of E12.5-E18.5 BrdU+ cells and E14.5-E18.5 BrdU+ cells was compared between WT mice (WT) and *Nsun5*-KO mice (KO). Representative images of immunostaining for BrdU in E18.5 cerebral cortices. Scale bars, 50 μm. Bar graphs (in middle column) show the total number of E12.5-E18.5 BrdU+ cells per section (upper panel) and E14.5-E18.5 BrdU+ cells (bottom panel). *P < 0.05 vs. WT mice. The percentage (%) of E12.5-E18.5 BrdU+ cells (upper panel) or E14.5-E18.5 BrdU+ cells (bottom panel) located in the upper-layers, deeper-layers and subcortical region was showed in the bar graphs (in right column). *P < 0.05 and **P < 0.01 vs. WT mice. **d** Representative images of cleaved caspase-3 immunostaining. Scale bars, 50 μm. Bar graph shows numbers of caspase-3+ cells (arrows) in E18.5 WT mice and *Nsun5*-KO mice. *P < 0.05 vs. WT mice

Next experiment was designed to evaluate whether the disrupted radial glial scaffolds in *Nsun5*-KO mice affects radial migration of cortical neurons. First, we examined the BrdU positive (BrdU+) cells in the E12.5 cerebral cortex which labels cells of the S-phase. The number of BrdU+ cells in *Nsun5*-KO mice was approximately equal to WT mice (*P* > 0.05, *n* = 6, Fig. 5a), indicating that the Nsun5 deficiency failed to alter the proliferation of RGCs. Subsequently, BrdU birth-dating was employed to elucidate the radial migration of cortical neurons [31]. Mice were injected with BrdU at E12.5 or E14.5, respectively, to label deeper-layer neurons (E12.5-E18.5 BrdU+ cells) and upper-layer neurons (E14.5-E18.5 BrdU+ cells) at E18.5 (*n* = 6 mice per experimental group). In comparison with WT mice, the total number of E12.5-E18.5 BrdU+ cells in the cerebral cortex of *Nsun5*-KO mice was unchanged (*P* > 0.05, *n* = 6, Fig. 5b), but the localization of E12.5-E18.5 BrdU+ cells in the deeper-layer was reduced by approximately 16% (*P* < 0.05, *n* = 6), since more cells were accumulated in the subcortical region (*P* < 0.01, *n* = 6). On the other hand, the total number of E14.5-E18.5 BrdU+ cells in *Nsun5*-KO mice was less than that in WT mice (*P* < 0.05, *n* = 6, Fig. 5c). Similarly, the number of E14.5-E18.5 BrdU+ cells in the upper-layers was visibly reduced in *Nsun5*-KO mice (*P* < 0.01, *n* = 6), which was companied by a subcortical accumulation of E14.5-E18.5 BrdU+ cells (*P* < 0.05, *n* = 6). Using the cleaved caspase-3 immunostaining, we observed an increase in the numbers of cleaved caspase-3 positive (caspase-3+) cells in the subcortical region of E18.5 *Nsun5*-KO mice (*P* < 0.05, *n* = 6, Fig. 5d), indicating the apoptosis of E14.5-E18.5 BrdU+ cells.

### Nsun5 deficiency disturbs the laminar location of cortical neurons

**Fig. 6:**
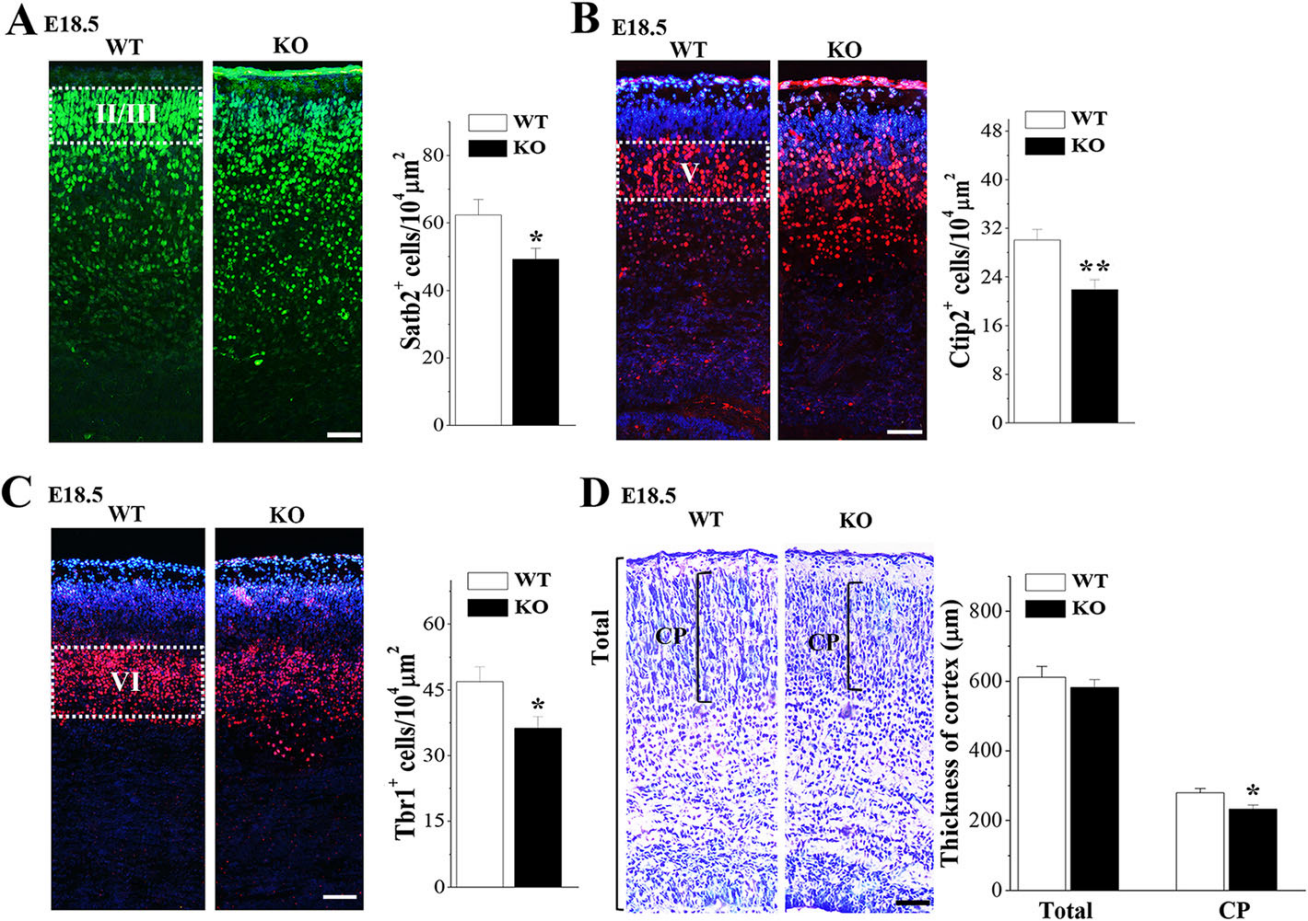
Nsun5 deficiency affects the laminar organization of cortical neurons. **a-c** Representative images of immunostaining for Tbr1, Ctip2 with DAPI or Stab2 in E18.5 WT mice (WT) and *Nsun5*-KO mice (KO). Scale bars, 50 μm. Bar graphs show the numbers of Tbr1+ cells in layer VI, Ctip2+ cells in layer V and Stab2+ cells in layers II/III. *P < 0.05 and **P < 0.01 vs. WT mice. **d** Coronal sections of cerebral cortices stained with toluidine blue in E18.5 WT mice and *Nsun5*-KO mice. Scale bars, 50 μm. Bar graph shows the total cortical thickness and CP. *P < 0.05 vs. WT mice

To determine further the effects of the Nsun5 deficiency on the lamination of upper-layer and deeper-layer neurons, layer-specific markers, including the anti-Tbr1 and anti-Ctip2 antibodies that label deeper-layer neurons and the anti-Satb2 antibody that labels upper-layer neurons were used (*n* = 6 mice per experimental group). In E18.5 WT mice, the majority of Tbr1 positive (Tbr1+) cells (Fig. 6c) and Ctip2 positive (Ctip2+) cells (Fig. 6b) had arrived at the deeper-layers of the CP, and Satb2 positive (Satb2+) cells were positioned in the upper-layers of CP (Fig. 6a). In contrast, the deeper-layer neurons and upper-layer neurons in E18.5 *Nsun5*-KO mice had not migrated into their appropriate lamina and exhibited a disordered distribution in the lower area, mainly in the intermediate zone (IZ). Compared with those in WT mice, the numbers of Tbr1+ cells in cortical layer VI (*P* < 0.05, *n* = 6) and Ctip2+ cells in cortical layer V (*P* < 0.01, *n* = 6) or the number of Satb2+ cells in cortical layers II/III (*P* < 0.05, *n* = 6) were significantly reduced in *Nsun5*-KO mice. Although the thickness of total cortical wall in E18.5 *Nsun5*-KO mice was not significantly altered (*P* > 0.05, *n* = 6; Fig. 6d), the stenosis of the CP was obvious (*P* < 0.05, *n* = 6).

### Possible mechanisms underlying Nsun5 deletion-impaired glial scaffolds

**Fig. 7:**
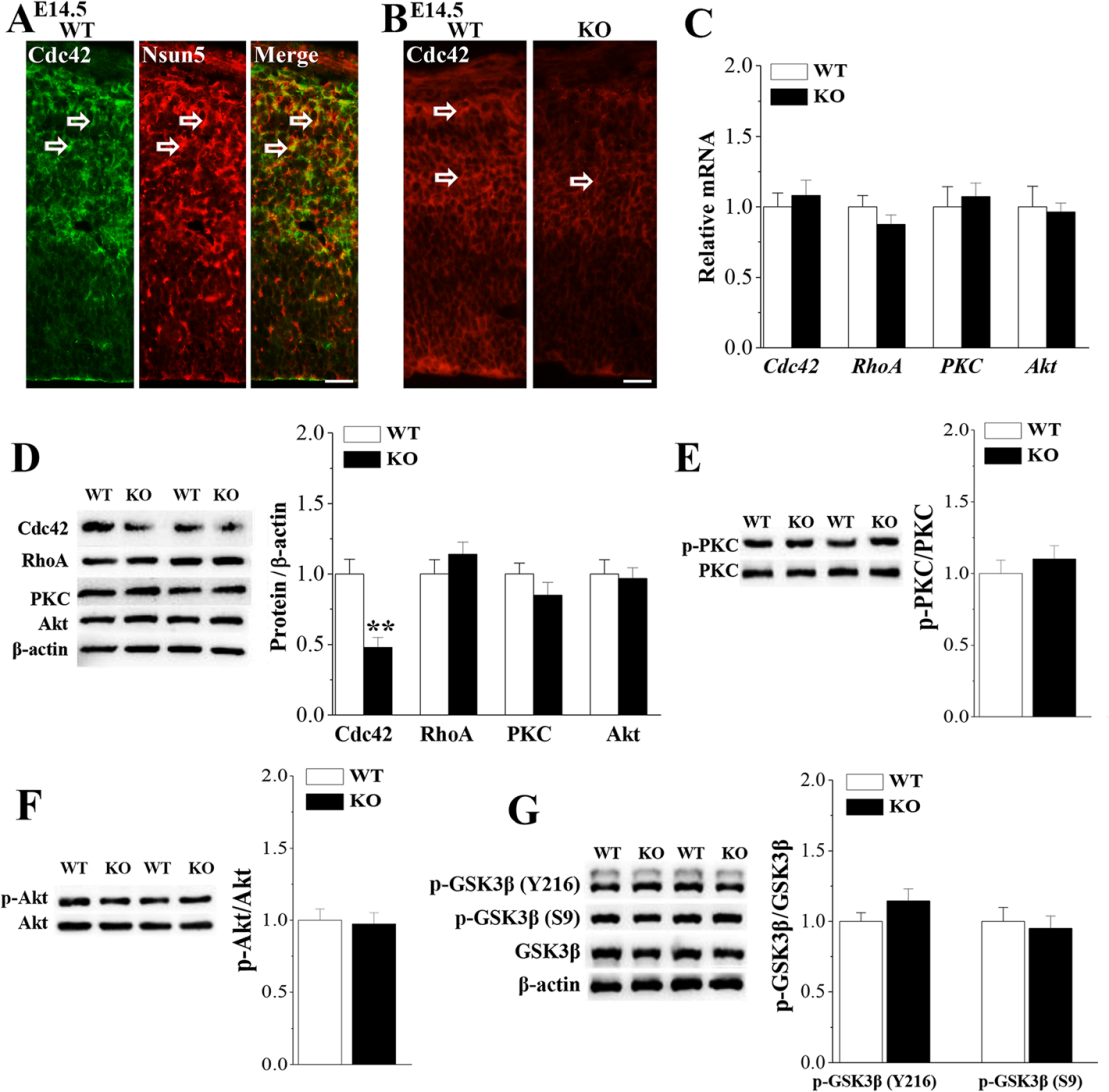
Nsun5 deficiency reduces the Cdc42 expression. **a** Representative images of double immunostaining for Cdc42 (green) and Nsun5 (red) in E14.5 WT mice (WT). Scale bars, 50 μm. **b** Representative images of Cdc42 immunoreactivity (open arrows) in E14.5 WT mice (WT) and *Nsun5*-KO mice (KO). Scale bars, 50 μm. **c** Levels of *Cdc42*, *RhoA*, *PKCζ* and *Akt* mRNA. **d** Levels of Cdc42, RhoA, PKCζ and Akt protein in cerebral cortex of WT mice and *Nsun5*-KO mice. **P < 0.01 vs. WT mice. **e-g** Levels of PKC (p-PKC), Akt (p-Akt), GSK3β at Y216 (p-GSK3β-Y216) and GSK3β at S9 (p-GSK3β-S9) phosphorylation

The development of radial glial scaffold during corticogenesis depends on the dynamic modulation of cytoskeletal and molecular polarity [32]. Polarized expression of cell polarity regulator Cdc42 and PKCζ in RGCs regulates glial end-feet activities and inter-radial glial interactions [33]. In addition, the genetic deletion of the small GTPase RhoA in the developing cerebral cortex results in migration disorders [34]. As shown in Fig. 7a, the Cdc42 protein was mainly located in the radial glial scaffolds of RGCs and overlapped perfectly with Nsun5 in cerebral cortex of E14.5 WT mice. Notably, the immunoreaction of Cdc42 in E14.5 *Nsun5*-KO mice was weaker than that in WT mice (Fig. 7b). Moreover, western blot analysis showed that the level of Cdc42 protein in *Nsun5*-KO mice was reduced compared to WT mice (*P* < 0.01, *n* = 6, Fig. 7d). However, the level of *Cdc42* mRNA was unchanged in *Nsun5*-KO mice (*P >* 0.05, *n* = 6; Fig. 7c). In contrast, neither the levels of RhoA, PKCζ and Akt protein (*P >* 0.05, *n* = 6) nor the levels of *RhoA*, *PKCζ* and *Akt* mRNA (*P >* 0.05, *n* = 6) were altered in *Nsun5*-KO mice. In addition, *Nsun5*-KO mice did not show the changes in the levels of PKCζ (*P >* 0.05, *n* = 6, Fig. 7e) and Akt expression or phosphorylation (*P >* 0.05, *n* = 6, Fig. 7f). The appropriate regulation of GSK3β is required to maintain the overall polarity of the radial glial scaffold [33]. However, the levels of phospho-GSK3β at Tyr216 (*P >* 0.05, *n* = 6, Fig. 7g) and at Ser9 (*P >* 0.05, *n* = 6) failed to be altered in *Nsun5*-KO mice.

## Discussion

The Nsun5 protein was selectively expressed in radial glial cells during embryonic cortex. The single-gene *Nsun5* knockout in mice disrupted the growth of radial glial scaffolds during corticogenesis to impede the radial migration of neocortical neurons, resulting in confused positioning of upper-layer and deeper-layer neurons and the morphological abnormalities in pyramidal neurons. Nsun5 is heterozygous in patients WBS. Not only *Nsun5* knockout homozygous but also heterozygous deletion of *Nsun5* in mice (*Nsun5*+/− mice) caused the phenotype of spatial cognitive defects [13]. Similarly, we in the present study observed that the thickness of the cortical plate in *Nsun5*+/− mice (Additional file 1: Figure S1A&B) and the apical dendrite of pyramidal cells in the layer V of *Nsun5*+/− mice were less than those in WT mice (Additional file 1: Figure S1C). The findings in the present study provided the first in vivo evidence that the transiently high expression of Nsun5 during corticogenesis is essential for the development of cerebral cortex.

In the embryonic neocortex, the Nsun5 protein was expressed at relatively high levels as early as E12.5, reached peak levels at E14.5. The Nsun5 protein in E14.5 neocortex was identified in the bodies of Sox2+ RGCs and processes of nestin+ RGCs^.^ Moreover, we observed the co-localization the Nsun5 protein and the cell polarity regulator Cdc42 in the CP and MZ of E14.5 neocortex. The thickness of the VZ, which reflects the size of the RGC pool, increases from E11.5 to E13.5, stays unchanged at E13.5-E14.5, and decreases from E14.5 to E16.5 [35]. The loss of Nsun5 has been reported to reduce the replicative lifespan of yeast mother cells [20]. Our results indicate that the deletion of Nsun5 in developing cerebral cortex likely does not affect the proliferation of RGC, since the number of BrdU+ cells in the VZ of E12.5 *Nsun5*-KO mice was not changed. An earlier study [36] reported that the loss of Cdc42 resulted in the gradual conversion of apical VZ progenitors to basal SVZ progenitors, indicating that Cdc42 is crucial for the maintenance of VZ progenitors. The Cdc42 deletion is reported to cause a displacement of RGCs within the proliferative niche [33]. Although the expression level of Cdc42 was reduced in the cerebral cortex of E14.5 *Nsun5*-KO mice, the number of Sox2+ RGCs and Tbr2+ IPCs in the VZ did not differ significantly from WT mice.

Layer formation in the developing cerebral cortex requires the movement of neurons from their site of origin to their final laminar position. The early-born neurons occupy the deep layers, while later-generated cortical plate cells migrate past deeper-layer neurons and settle in more superficial layers. Several studies have confirmed that the RGCs, which reside in the VZ and extend a fibre to the pial surface [29], serve as guides along which the neurons migrate to reach the correct lamina of the cortical plate, where they will become pyramidal cells in the adult cortex [37, 38]. Although the number of RGCs was unchanged in *Nsun5*-KO mice, the basal radial processes were distinctly reduced at E14.5-E18.5. Furthermore, the basal processes in *Nsun5*-KO mice were truncated, and did not extend perpendicularly to the pial surface. Thus, it is indicated that the impaired radial glial scaffolds in *Nsun5*-KO mice failed to provide guidance for migration of cortical neurons. Indeed, the idea is supported by the experimental results that the upper-layer and deeper-layer cortical neurons did not migrate into the appropriate lamina and arrested just below the cortical plate in E18.5 *Nsun5*-KO mice, resulting in the thinning of layers II-V in the cerebral cortex of PND10 *Nsun5*-KO mice. Two general modes of migration have previously been defined in the developing cerebral cortex: locomotion and nuclear translocation. The radial glia-guided locomotion involves the movement of the entire cell. During nuclear translocation, the cell first extends a leading process in the direction of migration, and then moves the nucleus to reach destination. The upper-layer cortical neurons migrate by radial glia-guided locomotion [39], whereas the deeper-layer cortical neurons migrate by a radial glia-independent manner [40]. Nadarajah et al. have demonstrated that locomotion and nuclear translocation are not cell-type specific and are responsible for the radial migration of cortical neurons. Although at early ages some cells may move by translocation only, locomoting cells also translocate once their leading process reaches the marginal zone [41, 42].

The final laminar positioning of cortical neurons is co-regulated by cell type- and layer-specific transcription factors that play concomitant roles in determining the molecular identity and axonal connectivity of these neurons. In the developing neocortex, disrupted neuronal migration affects the net forward extension of their axons, leading to a termination of axonal growth [43]. Indeed, we observed considerable deficits in the axonal growth and branching of pyramidal cells in postnatal cerebral cortex of *Nsun5*-KO mice. Importantly, the number of upper-layer neurons (E14.5-E18.5 BrdU+ cells) was reduced in *Nsun5*-KO mice. The RGCs, as a source of neurons and IPCs, is a key step in determining overall neuronal production [44]. The number of Tbr2+ IPCs in the SVZ of *Nsun5*-KO mice was not reduced, indicating that the reduction of upper-layer neurons is unlikely due to deficit in the neuronal production of RGCs. In the neocortex of *Nsun5*-KO mice, we observed an obvious increase in the number of apoptotic cells. The findings would help explain the possible mechanisms underlying Nsun5 deletion-impaired development of cerebral cortex: the Nsun5 deficiency in RGCs disrupts the growth of radial glial scaffolds; the radial migration of cortical neurons does not progress smoothly from the ventricles to the cortical plate, resulting in an abnormal laminar organization that affects the development of pyramidal cells and causes neuronal death. The pre-mature loss of cortical neurons in the embryonic cortex can produce a smaller neocortex. The Nsun5 deficiency caused the thinning of the cortical plate, whereas the total cortical thickness or the size of entire brain in *Nsun5*-KO mice did not exhibit obvious reduction in comparison with WT mice. A possible reason might be an expansion of the subcortical region in *Nsun5*-KO mice. Accumulating evidence suggests that loss-of-function mutations in genes encoding cytoskeletal regulators can cause the subcortical accumulation of multipolar cells since the neuronal migration are impaired, leading to the malformation periventricular heterotopias [43, 45, 46]. The genetic deletion of the doublecortin gene in mice causes the pre-mature termination of migration of many neurons to form subcortical heterotopias within the IZ [46].

The function of radial glia during corticogenesis depends on the dynamic modulation of cytoskeletal and molecular polarity [32, 47]. Cdc42 localizes to dynamically active regions of radial glia in the developing cerebral cortex. Disruption of Cdc42 activity altered polarized growth of radial glial cells, radial glial end-feet activities and inter-radial glial interactions [33]. Cdc42 is required to maintain the apico-basal polarity and adherens junctions [36, 48]. A critical finding in the present study is that the Cdc42 protein in *Nsun5*-KO mice was reduced, which affects the growth and function of radial glial scaffold. The conditional inactivation of GSK3β in radial progenitors has been reported to disrupt the growth of radial processes and the migration and placement of neurons [33]. *Nsun5*-KO mice did not show the changes in the expression and phosphorylation of GSK3β. Nsun5 is known to be a static molecular machine executing translation, the ribosome exhibits functional diversity by modifying a single rRNA nucleotide, resulting in an alteration of rRNA-mediated translational regulation [20]. It is proposed that the Nsun5-dependent methylation of rRNA is important for a regulation of ribosomal function. Many mRNAs have short upstream open reading frames within their 5’UTRs, which are known to modulate gene expression at the translational level [49, 50]. The decrease in the Cdc42 protein in *Nsun5*-KO mice was not associated with changes in the level of *Cdc42* mRNA. Thus, the Nsun5 deficiency is highly likely to affect the Cdc42 expression at the translational level. Certainly, study of the molecular mechanisms underlying the Nsun5 deficiency-reduced translation of Cdc42 should be an interesting subject for future work.

The Nsun5 protein is selectively expressed in RGCs at embryonic stages. The results obtained from *Nsun5*-KO mice will bring new insights into the critical role of the RNA methyltransferase Nsun5 in the development of the cerebral cortex. Although much more work needs to be performed, the Nsun5 deficiency might be a major contributing factor to the neuro-developmental phenotype of WBS, which might be associated with the cognitive behavioral profile of WBS patients.

## Materials and methods

### Generation and identification of Nsun5 null mice

The procedures involving animals and their care were conducted in accordance with the ARRIVE guidelines of Laboratory Animal Care [51]. All animal experiments were approved by the Institutional Animal Care and Ethical Committee of the Nanjing Medical University (No. 2014–153) and were performed in accordance with the guidelines of the Laboratory Animal Research Institute for Experimental Animals of Nanjing Medical University. All efforts were made to minimize animal suffering and to reduce the number of animals used. The mice were maintained under constant environmental conditions (temperature of 23 ± 2 °C, humidity of 55 ± 5%, and a 12:12-h light/dark cycle) in the Animal Research Center of Nanjing Medical University with free access to food and water. The *Nsun5*-KO mouse was generated by CRISPR/Cas9 genome editing. The oligos used to generate sgRNA expression plasmids were annealed and cloned into the BsaI sites of pUC57-sgRNA (Addgene 51,132) as previously described [28]. The following oligo sequences are used: sgRNA1-sense: TAGGCCCAGCAGAGCCTTCCAT; sgRNA1-antisense: AAACatggaaggctctgctggg; sgRNA2-sense: TAGGctgagctggcccgactca; and sgRNA2-anntisense: AAACTGAGTCGGGCCAGCTCAG. Founder mice were back-crossed onto the C57BL/6 J background. Homologous *Nsun5* (*Nsun5*−/−) mice (hereafter referred to as *Nsun5*-KO mice) were then obtained by mating heterozygous *Nsun5* (*Nsun5*+/−) mice.

### Experimental design

The morning of vaginal plug detection was designated embryonic day (E) 0.5 and the day of birth was considered postnatal day (PND) 0. The mice from E10.5 to PND10 were randomly divided into 5 experimental groups. First, we observed the cortical thickness and laminar organization, and the morphological alteration of the pyramidal neurons in PND10 *Nsun5*-KO mice. Subsequently, we determined the dynamic expression of Nsun5 and the distribution of Nsun5 positive (Nsun5+) cells in developing cerebral cortex from E10.5 to PND10. After the characteristics of Nsun5+ cells were identified, we investigated the effects of Nsun5 deficiency on growth of radial glial scaffold, proliferation, differentiation and transformation of RGCs. We further examined the influence of Nsun5 deficiency on radial migration and laminar location of cortical neurons in the development of cerebral cortex. Finally, we explored the mechanisms underlying Nsun5-regulated growth of radial glial scaffold.

### Antibodies

The following commercially available antibodies were used: Rabbit anti-Nsun5 (15449–1-AP, Proteintech Group Inc., China; Western blot/1:300, IF/1:100), rabbit anti-Cdc42 (2466, Cell Signaling Technology Inc., Boston, MA, USA; 1:1000), rabbit anti-phosphorylated glycogen synthase kinase 3β (p-GSK3β, Ser9, 9322, Cell Signaling Technology; 1:1000), rabbit anti-phosphorylated GSK3β (Tyr216, sc-11,758, Santa Cruz Biotechnology Inc.; 1:1000), rabbit anti-GSK3β (ab32391, Abcam, Cambridge, United Kingdom; 1:1000), rabbit anti-RhoA (10749–1-AP, Proteintech Group; 1:1000), rabbit anti-Akt (4691, Cell Signaling Technology; 1:1000), rabbit anti-phosphorylated Akt (4060, Cell Signaling Technology; 1:1000), rabbit anti-PKCζ (9368, Cell Signaling Technology; 1:1000), rabbit anti-phosphorylated PKCζ (9378, Cell Signaling Technology; 1:1000), mouse anti-β-actin (3700, Cell Signaling Technology; 1:1000), mouse anti-BrdU antibody (MAB4072, Millipore, Billerica, MA, USA; 1:1000), mouse anti-nestin (Rat-401, Developmental Studies Hybridoma Bank; 1:150), rabbit anti-BLBP (ab32423, Abcam; 1:1000), mouse anti-Sox2 (sc-365,823, Santa Cruz Biotechnology; 1:100), rat anti-Ctip2 (ab18465, Abcam; 1:500), rabbit anti-Tbr1 (ab31940, Abcam; 1:1000), mouse anti-Satb2 (ab51502, Abcam; 1:200), rabbit anti-cleaved caspase-3 (ab2302, Abcam; 1:300), rabbit anti-Tbr2 (ab23345, Abcam; 1:500) or chicken anti-Tbr2 (AB15894, Millipore; 1:1000). The secondary antibodies conjugated with Alexa fluorophores 488 or 555 (Invitrogen; 1:500) and the biotinylated goat anti-mouse secondary antibody (Santa cruz biotechnology; 1:200) were directed against the IgGs of the primary antibody species.

### Immunohistochemistry examination

Mice older than E12.5 were deeply anesthetized with isoflurane and perfused with cold 0.1 M PBS followed by 4% paraformaldehyde (PFA) for 6 h at 4 °C and transferred into 30% sucrose solution for cryoprotection. The coronal sections were cut at 10 or 70 μm, respectively, using a freezing microtome (Leica, Nussloch, Germany). The sections were mounted on gelatin-coated slides and allowed to dry over-night at room temperature.

#### Toluidine blue staining and Nissl staining

After gradient dehydration, the 10 μm sections were performed the toluidine blue staining and the 70 μm sections were stained with Nissl solution using standard protocols [52].

#### Immunofluorescence staining

Twelve histological sections per mouse were used for immunostaining of each antibody. The immunofluorescence staining was performed as previously described [53]. The sections were blocked with 1% bovine serum albumin (BSA) for 60 min at 4 °C and incubated with the primary antibodies against Nsun5, nestin, BLBP, Sox2, Ctip2, Tbr1, Satb2, Tbr2, cleaved caspase-3 or Cdc42 overnight at 4 °C. For double immunofluorescence staining, sections were incubated with two primary antibodies: Nsun5 and Sox2, Nsun5 and nestin, Nsun5 and Ctip2, Nsun5 and Satb2, Nsun5 and Cdc42. After washing, sections were incubated with the appropriate secondary antibodies for 2 h at room temperature. Sections were incubated with DAPI (Sigma) for 5 min.

#### BrdU immunostaining and birth-dating experiments

The thymidine analogue BrdU (Sigma-Aldrich, St. Louis, MO) was intraperitoneally injected into pregnant females at a concentration of 50 mg/kg body weight. For acute BrdU labeling at E12.5, embryonic brains were harvested 30 min after a single injection of BrdU. For birth-dating experiments, BrdU was injection at E12.5 or E14.5, and then brains were fixed with 4% PFA at E18.5 mice to examine E12.5-E18.5 BrdU+ cells (deeper-layer neurons) and E14.5-E18.5 BrdU+ cells (upper-layer neurons) as previously described [31]. The coronal sections (10 μm) were acid-treated with 2 M HCl for 30 min at 37 °C, and then incubated in primary antibody of anti-BrdU overnight at 4 °C. Immunoreactivities were visualized with avidin-biotin horseradish peroxidase complex (ABC Elite; Vector Laboratories, Inc., Burlingame, CA, USA) using 3,3′-diaminobenzidine as chromogen.

#### Morphometric analysis and statistics

(1) Images of Nissl staining were obtained on a conventional light microscope (Olympus DP70, Tokyo, Japan) by a stereological system, consisting of a CCD camera (Olympus DP70), a motorized specimen stage for automatic sampling, and a computer running Microbrightfield Stereo Investigator software (MicroBrightField, Williston, USA). Layers I, II/III, V, and VI were discriminated using multiple criteria. Layer VI was clearly distinguishable from layer V based on the cell orientations. In layer V, most pyramidal cells were oriented perpendicular to the pial surface and the external capsule. Layer II/III pyramidal cells had a short apical dendrite and extensive basilar dendrites. In contrast, layer V pyramidal cells had a long apical dendrite and less extensive basilar dendrites. Z-stack images were taken in the cortex at 40 ×. We used the Neurolucida Neuron Tracing Software to trace the apical dendrite of layer V pyramidal cells [54] to measure the length of the apical dendrite. Image analysis was completed through ImageJ software. (2) Images of immunofluorescence staining were captured using a fluorescence microscope (Olympus DP70, Tokyo, Japan). At least six embryos were analyzed for each experimental group of morphometric analysis. Ten matching sections from both hemispheres of each brain were used for the measurements and comparisons. The Sox2+ cells, Tbr2+ cells and cleaved caspase-3+ cells in each radial segment were 200 μm in width and spanned from the ventricular surface to the pia [30] were counted using the manual tag function of Image Pro-Plus 6 (Media Cybernetics) by an experimenter who was blinded to the mouse genotype. (3) For the cell proliferation assay, the number of BrdU+ cells in each radial unit was counted [31]. (4) For the radial migration analysis of neurons, the distribution of E12.5-E18.5 BrdU+ cells (deeper-layer neurons) and E14.5-E18.5 BrdU+ cells (upper-layer neurons) was analyzed in the different compartments of the cortex. The cortex was divided into ten bins of with 300 μm in width. Subsequently, bins 1–3, 4–6 and 7–10 were again grouped as upper-layer, deeper-layer (middle region) and subcortical region (bottom region), respectively. The percentage of BrdU+ cells in each layer was determined, and results were plotted as histograms.

### Western blot analysis

The cerebral cortices were homogenized in 200 μl of Tris buffer (pH 7.4) containing 10% sucrose, phosphatase inhibitors and protease inhibitors (Complete; Roche Diagnostics), sonicated and stored at − 80 °C until use. Protein concentrations were quantified using the BCA assay (Pierce). Equal amounts of protein were separated by SDS-polyacrylamide gel electrophoresis and transferred to PVDF membranes. Membranes were blocked with 5% non-fat milk in Tris-buffered saline (TBS)/Tween-20 and then incubated with antibodies against Nsun5, Cdc42, RhoA, p-Akt, p-PKC or p-GSK3β (Ser9 or Tyr216) at 4 °C for 24 h. Appropriate HRP-conjugated secondary antibodies were incubated with the membranes for 1 h at room temperature and signals were visualized using an enhanced chemiluminescence detection kit (ECL, Millipore). Following visualization, the blots were stripped with stripping buffer for 15 min and then incubated with antibodies against Akt, PKC, GSK3β or β-actin at 4 °C for 24 h. Western blot bands were scanned and analyzed using the ImageJ software package (NIH).

### Reverse transcription-polymerase chain reaction (RT-PCR)

Embryos were moved from pregnant females that had been anesthetized with CO_2_ and placed on ice. The dorsal cerebral cortices (E14.5-PND10) were dissected, or the total telencephalon was removed (E10.5-E12.5). The tissue was immediately transferred to Trizol reagent (Invitrogen, Camarillo, CA) and processed for total RNA isolation according to the manufacturer’s protocol. Then, the total RNA was reverse-transcribed into cDNAs using a Prime Script RT reagent kit (Takara, Japan) for quantitative PCR (ABI Step One Plus, Foster City, CA) in the presence of a fluorescent dye (SYBR Green I; Takara). The relative expression of genes was determined using the 2-ΔΔct method with normalization to *GAPDH* expression. The primers for RT-PCR were designed based on published mouse gene sequences. The primers for *Nsun5*, *Cdc42*, *RhoA*, *PKC*, *Akt* and *GAPDH* are listed in Table 1 [29, 31, 55].

**Table 1:** Primers for quantitative real-time PCR

	Forward	Reverse
*Nsun5*	GAGGGAAGGGTGGATAAGG	GGCACGATGCGGATGTAG
*Cdc42*	GTTGGTGATGGTGCTGTTG	CTGTGGATAACTTAGCGGTCG
*RhoA*	CATTGACAGCCCTGATAGTT	TCGTCATTCCGAAGGTCCTT
*PKC*	ACCCTCGTAGAGAAGCGTGT	TGAAAGTGGAGTGAAGCTG
*GAPDH*	TGGGTGTGAACCACGAG	ACCACAGTCCATGCCATCAC

### Statistical analyses

Data were retrieved and processed using MicroCal Origin 9.2 software (Origin Lab, Northampton, MA, USA). Group data are presented as means ± standard errors of the means (SEM). All statistical analyses were performed using SPSS software, version 18.0 (SPSS Inc., Chicago, IL, USA). Differences between means were analyzed using one way Analysis of Variance (ANOVA) and Student’s t test. *P < 0.05, **P < 0.01.

## Additional file

Additional file 1: **Figure S1.** Nsun5 deficiency impairs development of cerebral cortex. **A** Representative images of cerebral cortex stained with toluidine blue in WT mice and heterozygous deletion of Nsun5 (Nsun5+/-) mice. Scale bars, 100 μm. **B** Bar graph shows the thickness of layers I-VI. *P < 0.05 vs. WT mice (Student's t test). **C** Higher power views of the boxed areas in the layer V. Scale bars, 50 μm. Representative images of pyramidal cells (arrows) are shown in the bottom right insets. The open arrows indicate the apical dendrite of pyramidal cells. Bar graph shows the length of the apical dendrite in WT mice and Nsun5+/- mice. *P < 0.05 vs. WT mice (Student's t test). (DOC 2565 kb)

## Data Availability

All data generated or analyzed during this study are included in this published article.

## Abbreviations

BLBP: Brain lipid binding protein
BM: Basement membrane
BSA: Bovine serum albumin
DCX: Doublecortin
IPC: Intermediate progenitor cell
IZ: Intermediate zone
PND: Postnatal day
RGC: Radial glial cell
SVZ: Subventricular zone
VZ: Ventricular zone
WBS: Williams-Beuren syndrome
