# Supplementary material for: Expression of the RNA methyltransferase Nsun5 is essential for developing cerebral cortex
Source: Mol Brain. 2019 Aug 28;12:74. doi: 10.1186/s13041-019-0496-6 (PMC6714381; doi:10.1186/s13041-019-0496-6)
Supplement: Supplementary file 1 — Figure S1. Nsun5 deficiency impairs development of cerebral cortex. A Representative images of cerebral cortex stained with toluidine blue in WT mice and heterozygous deletion of Nsun5 (Nsun5+/-) mice. Scale bars, 100 μm. B Bar graph shows the thickness of layers I-VI. *P < 0.05 vs. WT mice (Student's t test). C Higher power views of the boxed areas in the layer V. Scale bars, 50 μm. Representative images of pyramidal cells (arrows) are shown in the bottom right insets. The open arrows indicate the apical dendrite of pyramidal cells. Bar graph shows the length of the apical dendrite in WT mice and Nsun5+/- mice. *P < 0.05 vs. WT mice (Student's t test). (DOC 2565 kb) [file 13041_2019_496_MOESM1_ESM.doc]

**Supplemental information**

***Molecular Brain***

**Expression of the RNA methyltransferase Nsun5 is essential for developing cerebral cortex**

#Peipei Chen^1,2^, #Tingting Zhang^1,2^, Zihao Yuan^2^, Bin Shen^1,*^ and Ling Chen^1,2, *^

^1^State Key Laboratory of Reproductive Medicine, ^2^Department of Physiology, Nanjing Medical University, Nanjing, 211166, China

Short title: Nsun5 deletion-impaired RGC development

*Correspondence author: Ling Chen, Ph.D. & M.D.

Address: State Key Laboratory of Reproductive Medicine, Department of Physiology, Nanjing Medical University, Tianyuan East Road 818, Nanjing, China.

Tel: +86-25-86869441, Fax: +86-25-86869441

E-mail: [lingchen@njmu.edu.cn](mailto:lingchen@njmu.edu.cn)

*Correspondence author: Bin Shen, Ph.D.

Address: State Key Laboratory of Reproductive Medicine, Nanjing Medical University, Tianyuan East Road 818, Nanjing, China.

Tel: +86-25-86869441, Fax: +86-25-86869441

E-mail:  [binshen@njmu.edu.cn](mailto:%09binshen@njmu.edu.cn)

Supplemental data, S-Figure 1

**
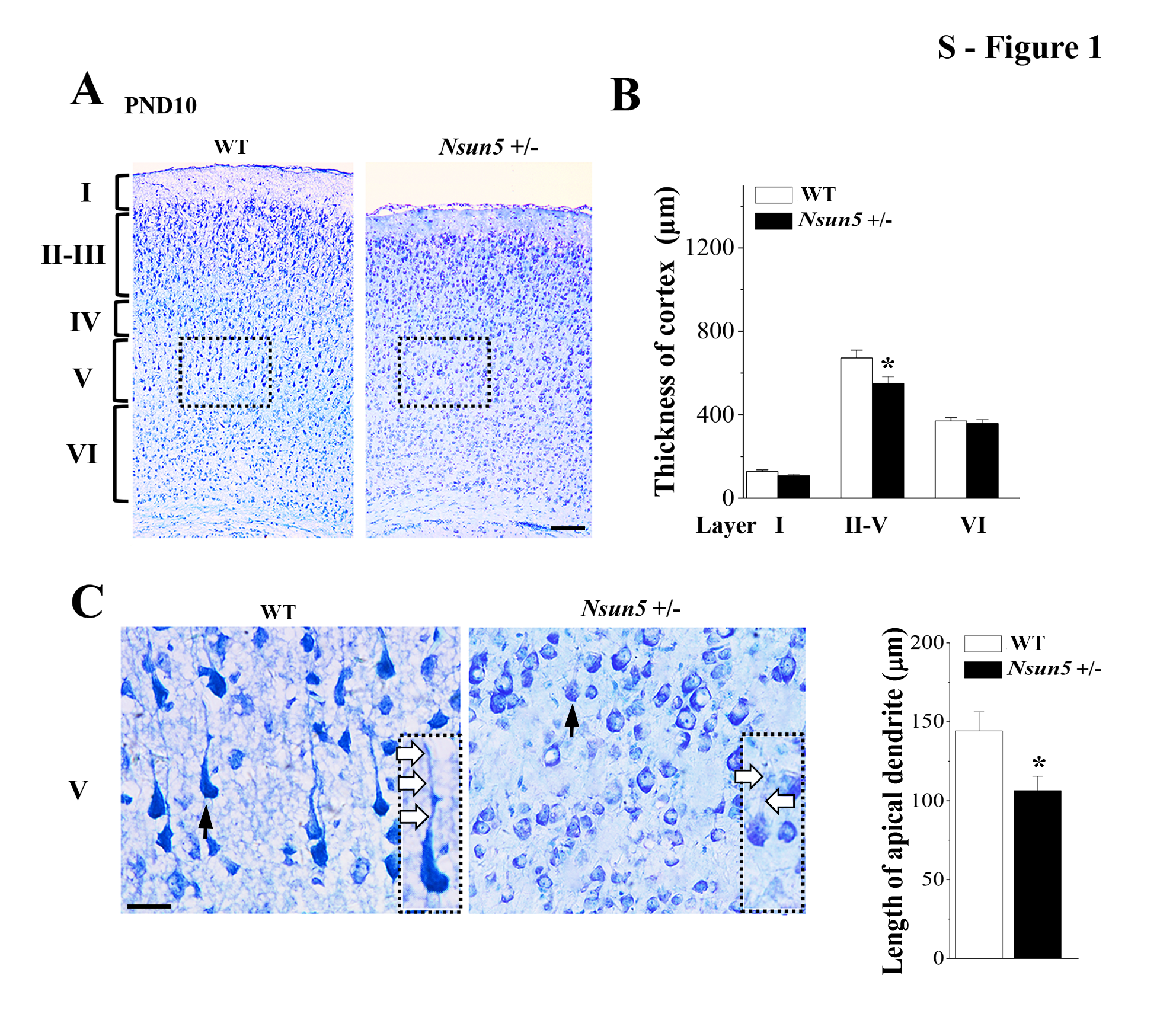
**

**Figure S1.** **Nsun5 deﬁciency impairs development of cerebral cortex**. (**A**) Representative images of cerebral cortex stained with toluidine blue in WT mice and heterozygous deletion of *Nsun5* (*Nsun5*+/-) mice. Scale bars, 100 μm. (**B**) Bar graph shows the thickness of layers I-VI. **P* < 0.05 *vs*. WT mice (Student's *t* test). (**C**) Higher power views of the boxed areas in the layer V. Scale bars, 50 μm. Representative images of pyramidal cells (arrows) are shown in the bottom right insets. The open arrows indicate the apical dendrite of pyramidal cells. Bar graph shows the length of the apical dendrite in WT mice and *Nsun5*+/- mice. **P* < 0.05 *vs*. WT mice (Student's t test).
